# Krenning score enhances integrated biomarker models for survival prediction in PRRT-treated GEP-NET: a retrospective cohort study

**DOI:** 10.1186/s40644-026-01113-w

**Published:** 2026-08-25

**Authors:** Christian A. Dascalescu, Felix L. Herr, Ricarda Ebner, Victoria Fusch, Moritz L. Schnitzer, Matthias P. Fabritius, Christine Schmid-Tannwald, Mathias J. Zacherl, Vera Wenter, Matthias Brendel, Adrien Holzgreve, Christoph J. Auernhammer, Christine Spitzweg, Thomas Knösel, Tanja Burkard, Jens Ricke, Johannes Rübenthaler, Maurice M. Heimer, Gabriel T. Sheikh, Rudolf A. Werner, Konrad Klimek, Clemens C. Cyran

**Affiliations:** 1 Department of Radiology, LMU University Hospital, LMU Munich, Marchioninistr. 15, 81377 Munich, Germany; 2 Department of Nuclear Medicine, LMU University Hospital, LMU Munich, Munich, Germany; 3 Department of Medicine IV, LMU University Hospital, LMU Munich, Munich, Germany; 4 Department of Pathology, LMU University Hospital, LMU Munich, Munich, Germany

**Keywords:** Gastroenteropancreatic neuroendocrine tumor, Peptide receptor radionuclide therapy, Integrated diagnostics, Krenning score, Overall survival

## Abstract

**Background:**

Purpose of this study is to evaluate the value of a routinely available baseline SSTR-PET imaging parameter, the Krenning score (KS), within integrated biomarker models for overall survival prediction in gastroenteropancreatic neuroendocrine tumor (GEP-NET) patients undergoing peptide receptor radionuclide therapy (PRRT).

**Methods:**

We retrospectively analyzed 178 patients with GEP-NET who underwent PRRT. At baseline, the KS, clinical, histopathological, and laboratory parameters were integrated and correlated with OS. OS predictors were identified using univariate Cox regression analysis and incorporated into multivariate models. Model performance was assessed using the concordance index (C-index) and Akaike information criterion (AIC).

**Results:**

In univariate analysis, the following parameters were significantly associated with shorter OS: KS 3 vs. KS 4 (p = 0.042), CgA > 155 ng/mL (p = 0.003), NSE > 35 ng/mL (p = 0.042), BMI < 18.5 kg/m² (p = 0.017), CRP > 0.5 mg/dL (p = 0.045), albumin < 4.1 g/dL (p = 0.017), and Hb < 12 g/dL (p = 0.017). The multivariate Cox regression model including KS, hemoglobin, and NSE showed the lowest AIC (C-index = 0.64, CI: 0.57–0.71; AIC = 527.60), while the model incorporating BMI, hemoglobin, and CgA demonstrated the highest C-index (C-index = 0.66, CI: 0.60–0.72; AIC = 582.82).

**Conclusion:**

Integrated baseline biomarker models combining clinical, laboratory, and imaging parameters may support overall survival risk stratification in GEP-NET patients undergoing PRRT. Incorporation of the routinely available Krenning score contributed to one of the best-performing parsimonious exploratory multivariable models, suggesting that accessible molecular imaging parameters may complement established baseline biomarkers. These exploratory findings require validation in larger, preferably multicenter, independent cohorts before clinical implementation.

**Clinical trial number:**

Not applicable.

**Supplementary Information:**

The online version contains supplementary material available at 10.1186/s40644-026-01113-w.

## Background

Gastroenteropancreatic neuroendocrine tumors (GEP-NET) represent a heterogeneous group of malignancies characterized by the overexpression of somatostatin receptors, particularly subtype 2 (SSTR2) [1, 2]. Somatostatin-receptor-targeted (SSTR-) PET/CT not only enables precise tumor localization but also plays a key role in guiding therapy selection for patients [3–5]. Peptide receptor radionuclide therapy (PRRT) has emerged as an established treatment for GEP-NET [6–8]. A prerequisite for effective therapy is the confirmation of sufficient tracer uptake on SSTR-PET scans, typically quantified using the modified Krenning score (KS), a visual, semi-quantitative grading system that compares tumor uptake with physiological uptake in the liver and spleen [9, 10].

Given the biological heterogeneity and longitudinal clonal evolution of GEP-NETs, prognostic assessment remains challenging in routine clinical practice. Accordingly, current clinical guidelines from the European Neuroendocrine Tumor Society (ENETS) and the North American Neuroendocrine Tumor Society (NANETS) underscore the importance of histopathological assessments, including tumor classification, differentiation, and proliferation indices, as well as baseline measurements of circulating biomarkers such as chromogranin A (CgA) and neuron-specific enolase (NSE) [11, 12]. In addition, consensus recommendations on biomarkers in neuroendocrine tumor disease emphasize the established clinical role of CgA and NSE in disease assessment and prognostication, while also acknowledging limitations related to assay variability and clinical confounders. The consensus panel further concluded that no single monoanalyte biomarker has yet proven sufficient for optimal clinical use [13].

In recent years, the concept of integrated diagnostics has gained traction in oncology. This approach combines available data from imaging, pathology, clinical evaluations, and laboratory tests to create a precise picture of patient disease status. In other cancer types, integrated diagnostics has proven valuable for prognostication and personalized therapy planning [14–16].

Numerous prognostic models in PRRT-treated NETs have focused either on PFS, interim data analyses, or complex combinations of imaging, functional, and laboratory parameters, which limits their applicability in routine practice [17–21]. Despite these efforts, data on simple and clinically interpretable multivariable baseline models that incorporate a readily available, semi-quantitative imaging metric such as the Krenning score in PRRT-treated NET patients remain limited [18, 22]. Therefore, we aimed to evaluate a clinically applicable multivariable prognostic model for overall survival in PRRT-treated GEP-NET patients that integrates a readily available baseline simple semi-quantitative imaging metric, represented by KS, alongside clinical, histopathological, and biochemical markers. We hypothesized that an integrated baseline model incorporating the KS may contribute prognostic information within simple, routinely applicable biomarker models and support baseline risk stratification in this PRRT-treated GEP-NET cohort.

## Methods

### Study design and patient population

We conducted a single-center retrospective investigation approved by the local institutional review board (approval number 19–027). Informed consent was waived due to the retrospective design. The analysis included patients with histologically confirmed GEP-NET who received PRRT at an ENETS-certified tertiary care center between 2012 and 2023. Treatment decisions were determined by a multidisciplinary tumor board (MDT). Inclusion criteria comprised patients with differentiated (G1/G2) NET who underwent baseline imaging with either [⁶⁸Ga]Ga-DOTA-TATE, [⁶⁸Ga]Ga-DOTA-TOC, [⁶⁸Ga]Ga-DOTA-NOC, or [¹⁸F]SiTATE PET/CT, followed by [¹⁷⁷Lu]Lu-DOTA-TATE PRRT. The interval from initial diagnosis to first PRRT was recorded, and time metrics for treatment cycles and subsequent follow-up imaging were documented.

### Clinical and laboratory data collection

Demographic and clinical data, including age, date of diagnosis, primary tumor localization, and prior oncologic interventions were extracted from electronic health records. Laboratory values were obtained from the institutional laboratory information system, and imaging data were retrieved from the institutional picture archiving and communication system (PACS). All data were collected and stored in pseudonymized form on secured institutional servers in accordance with local data protection regulations and institutional review board approval. A comprehensive set of clinical and laboratory parameters was recorded at baseline and included body mass index (BMI), histopathological proliferation index (e.g., Ki-67), and serum biomarkers such as C-reactive protein (CRP), albumin, leukocyte count, erythrocyte count, hemoglobin, thrombocyte count, NSE, and CgA. Information on prior oncologic treatment was recorded where available and included in additional sensitivity analyses to address potential treatment-related confounding.

### Imaging protocol and analysis

In the included patients, whole-body SSTR-PET/CT was performed using a Siemens Biograph mCT (*n* = 110), Siemens Biograph 64 (*n* = 58), or GE Discovery 690 (*n* = 10). PET acquisition was initiated 60–90 min after intravenous injection of the radiotracer. Four different SSTR-targeting radiotracers were used: [⁶⁸Ga]Ga-DOTA-TATE (*n* = 71), [⁶⁸Ga]Ga-DOTA-TOC (*n* = 70), [⁶⁸Ga]Ga-DOTA-NOC (*n* = 1), and [¹⁸F]SiTATE (*n* = 36), with a mean administered activity of 228 ± 36 MBq. Semi-quantitative SSTR expression was determined in a consensus-based manner by two board-certified hybrid imaging specialists (CCC and MPF), with 20 and 5 years of experience in hybrid imaging, respectively, using KS based on the lesion with the highest uptake [23]. Both readers reviewed the PET/CT images together, and final Krenning scores were assigned after discussion until consensus was reached.

### Outcome measures and statistical analysis

Baseline was defined as the last available clinical, laboratory, and imaging assessment prior to initiation of PRRT. OS was defined as the interval from baseline evaluation to death from any cause, with patients censored at the time of last follow-up if no event occurred. Statistical analyses were performed using R (version 4.3.0; R Foundation for Statistical Computing, Vienna, Austria). Data normality was assessed by Kolmogorov-Smirnov tests, guiding the choice between parametric and non-parametric comparisons. To ensure a robust and reliable analysis, missing data were addressed using multiple imputation via the mice package in R (Multivariate Imputation by Chained Equations). Under the hypothesis that data were missing at random (MAR), five imputed datasets were created using predictive mean matching. Each dataset was analyzed independently, and results were combined using Rubin’s rules to obtain pooled estimates [24]. Sensitivity analyses confirmed that the imputations did not meaningfully alter the primary study findings.

Survival analyses were initially conducted using univariate methods; Kaplan-Meier curves were generated and group differences were evaluated with the log-rank test. Subsequently, exploratory Cox proportional hazards regression models were developed to identify independent predictors of OS, incorporating variables that were significant in univariate testing. Thresholds were selected either on established cut-off values reported in the literature, standard reference ranges, or on those that showed the strongest correlation with OS. Survival predictors were examined through both univariate and multivariate Cox regression models. To define optimal prognostic thresholds, a data-driven cut-point analysis was conducted using the survminer package in R. This approach applies maximally selected rank statistics to identify the cut-off values that stratify OS best, as determined by the log-rank test. The thresholds yielding the highest discriminatory power within the cohort were selected and subsequently used in both univariate and multivariate survival analyses. To mitigate the risk of false positives due to multiple comparisons, p-values were adjusted using the Benjamini-Hochberg method, effectively controlling the false discovery rate [25]. To assess potential redundancy among predictors, multicollinearity was evaluated using generalized variance inflation factors. In addition, LASSO-penalized Cox regression was performed as a secondary sensitivity analysis to assess the robustness of variable selection and reduce the risk of overfitting. Model performance was compared using the Akaike Information Criterion (AIC) and Harrell’s concordance index, with lower AIC values and higher concordance indices indicating superior model fit. Deviance (ANOVA) tests were performed to assess incremental improvements when adding predictors, and the statistical significance of covariates in the final model was confirmed using the Wald test [26, 27]. To facilitate visualization of risk stratification, a multivariate model was translated into a simplified baseline risk score. Each adverse predictor was assigned one point, yielding an additive score that allowed patients to be categorized according to the number of baseline risk factors present. To address the potential influence of equal weighting, the simplified equal-weight score was additionally compared with a coefficient-weighted Cox-derived score.

Additional Cox regression analyses included prior chemotherapy as a covariate, and primary tumor site-specific analyses were performed for pNET and siNET, including comparisons of baseline characteristics and OS as well as interaction analyses evaluating whether the prognostic associations of the investigated baseline biomarkers differed between both tumor entities.

## Results

### Patient characteristics

A comprehensive overview of the demographic characteristics, clinical and laboratory parameters is provided in Table 1. All patients were treated with [¹⁷⁷Lu]Lu-DOTA-TATE following standard institutional protocols, typically consisting of four treatment cycles administered at eight-week intervals. 1-, 2-, 3- and 5-year OS of all patients was 99%, 89%, 80% and 63%, respectively. With a median follow-up of 60.8 months, median OS was recorded as 70.1 months (range, 7-145 months). Of the 178 patients included in the analysis, 82 (46%) experienced the event of death during follow-up, while 96 patients (54%) had not experienced death by the end of the observation period. Median OS was 63.5 months for patients with progressive disease assessed by a multidisciplinary tumorboard, compared to 94.1 months for those without.

**Table 1:** Clinical characteristics of study population

	*n* = 178
**Male to female**	108 / 70
**Mean age at baseline** mean (SD)	63.5 years (10.6)
**Times** mean (SD)	
Time since diagnosis to baseline	47 months (56)
**BMI (kg/m**^**2**^**)** mean (SD)	24.9 (4.3)
**Primary tumor location**. n (%)	
Small bowel	98 (55%)
Pancreas	37 (21%)
Cancer of Unknown Primary	21 (12%)
Ileocecal junction	11 (6%)
Large bowel / Rectum	9 (5%)
Other	2 (1%)
**Tumor grade**. n (%)	
G1	54 (30%)
G2	119 (67%)
Unknown	5 (3%)
**Metastases**. n (%)	
Liver	144 (81%)
Lymph nodes	108 (61%)
Mesenterium / Peritoneum	108 (61%)
Bone	105 (59%)
Other	24 (13%)
**Therapy Approach before** PRRT *. n (%)	
Surgical resection of primary tumor	105 (59%)
Long-acting somatostatin analogue	110 (62%)
Local ablative liver therapies	49 (28%)
Chemotherapy or everolimus	30 (17%)
Bone-targeted radiotherapy	6 (3%)

*some patients received more than one treatment modality before PRRT

SD = standard deviation; PRRT = peptide receptor radionuclide therapy; BMI = body mass index; NET = neuroendocrine tumor

### Univariate analysis

**Fig. 1:**
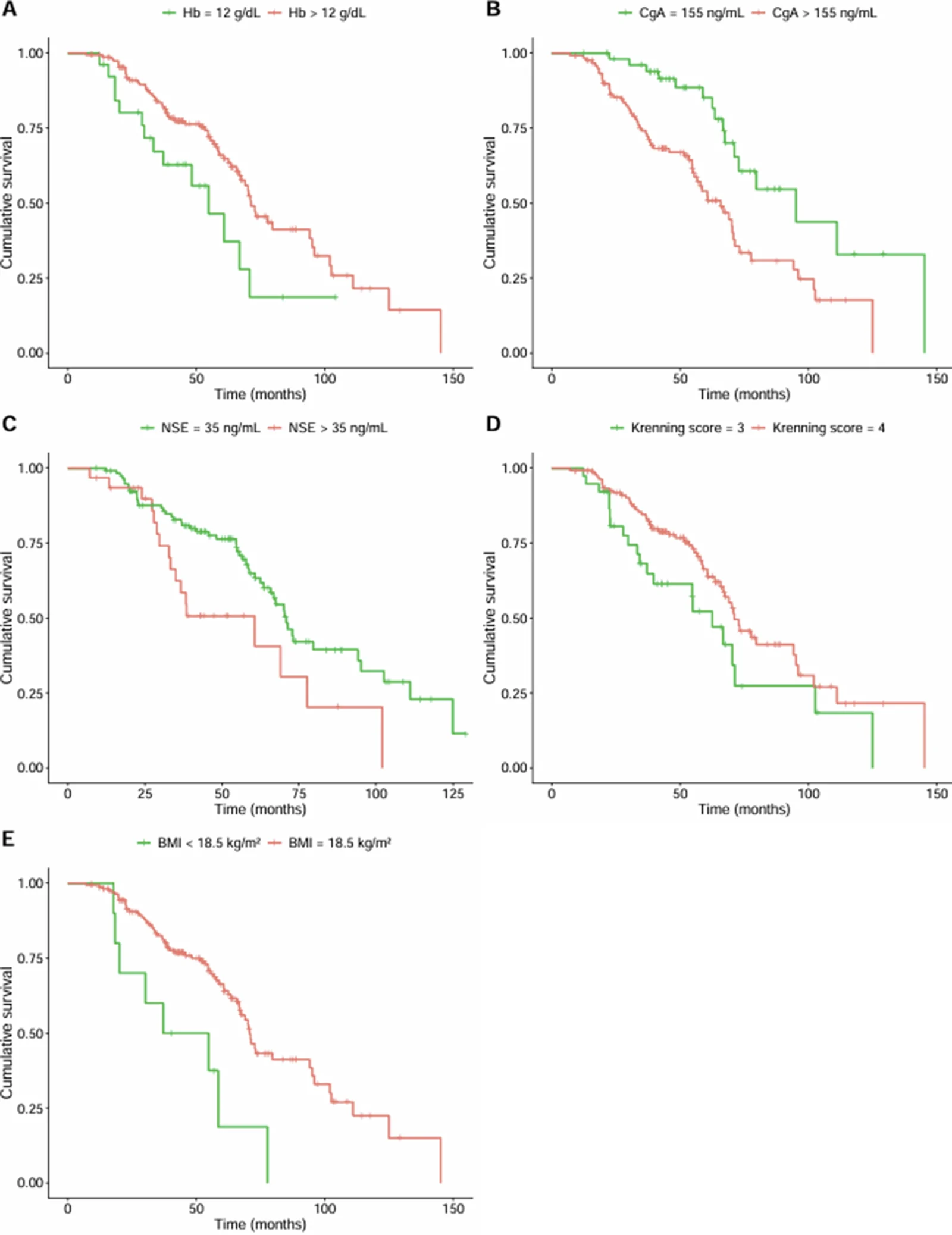
OS according to Krenning score (KS; **A**), BMI (**B**), hemoglobin (Hb; **C**), chromogranin A (CgA; **D**) and neuron-specific enolase (NSE; **E**) at baseline. Low KS (3 vs. 4), low BMI (< 18.5), low Hb (< 12 g/dL), and high CgA (> 155 ng/mL) and NSE (> 35 ng/mL) at baseline were each significantly associated with shorter overall survival compared with their respective reference groups

In univariate analysis, several baseline factors were significantly associated with shorter OS (Table 3). Patients with KS 3 vs. KS 4 (HR, 1.84; 95% CI, 1.11–3.04; *p* = 0.042), CgA > 155 ng/mL (HR, 2.31; 95% CI, 1.38–3.89; *p* = 0.003), NSE > 35 ng/mL (HR, 1.93;95% CI, 1.1–3.38; *p* = 0.042) showed shorter OS. Additionally, the following parameters were significantly correlated with shorter OS: BMI < 18.5 kg/m² (HR, 2.89; 95% CI, 1.27–3.22; *p* = 0.017), CRP > 0.5 mg/dL (HR, 1.84; 95% CI, 1.02–3.39; *p* = 0.045), albumin < 4.1 g/dL (HR, 2.09; 95% CI, 1.24–3.53; *p* = 0.017), and Hb < 12 g/dL (HR, 2.33; 95% CI, 1.29–4.22; *p* = 0.017) had significantly shorter OS (Fig. 1). Primary tumor location in the pancreas, Ki-67 index > 5%, leukocytes, erythrocytes and thrombocytes were not significantly correlated with OS (all; *p* > 0.05, Table 2). The largest improvements in model performance compared to the null model (AIC = 640.77) were observed for CRP (AIC = 452.62), albumin (AIC = 507.00), NSE (AIC = 535.23) and CgA (AIC = 588.54). These variables also demonstrated the highest concordance indices, with values of 0.56 for NSE, 0.59 for both CRP and albumin, and 0.61 for CgA, respectively.

**Table 2:** Univariate and multivariate analyses of factors contributing to OS. In the univariate analysis (log-rank-test), Krenning score, CgA > 155 ng/mL, NSE > 35 ng/mL, hemoglobin ≤ 12 g/dl, CRP levels > 0.5 mg/dL, and albumin < 4.1 g/dL (*p* < 0.05 for all) were associated with shorter OS

Variable	Univariate Analysis						Multivariate Analysis						
	Patients (*n*)	HR	(95% CI)	AIC	C-index	P*	HR	(95% CI)	Z	Wald	AIC (global)	C-index (global)	*P*
All patients	178										293.21	0.68	
Body mass index													
< 18.5	10	2.89	(1.27–3.22)	636.71	0.54	0.017	1.802	(0.60–5.46)	1.04	1.09			0.262
≥ 18.5	168												
Origin of primary													
Pancreas	36	1.14	(0.64–2.03)	642.57	0.53	0.707							
Other	137												
Ki-67 (%)													
> 5	60	1.43	(0.9–2.28)	640.42	0.54	0.176							
≤ 5	111												
Krenning													
3	38	1.84	(1.11–3.04)	637.75	0.56	0.042	0.921	(0.41–2.07)	-0.2	0.04			0.779
4	140												
CRP (mg/dL)													
> 0.5	43	1.86	(1.02–3.39)	452.62	0.59	0.045	1.166	(0.48–2.86)	0.34	0.11			0.712
≤ 0.5	81												
Albumin (g/dL)													
< 4.1	49	2.09	(1.24–3.53)	507	0.59	0.017	1.59	(0.76–3.31)	1.24	1.54			0.259
≥ 4.1	103												
Leukocytes (G/L)													
< 5	24	0.78	(0.39–1.56)	642.24	0.49	0.576							
≥ 5	154												
Erythrocytes (million/µL)													
< 4	25	1.74	(0.95–3.18)	639.94	0.54	0.111							
≥ 4	148												
Hemoglobin (g/dL)													
< 12	25	2.33	(1.29–4.22)	636.17	0.56	0.017	1.88	(0.74–4.75)	1.33	1.78			0.251
≥ 12	148												
Thrombocytes (g/L)													
< 200	49	1.07	(0.62–1.83)	642.72	0.51	0.813							
≥ 200	129												
NSE (ng/mL)													
> 35	52	1.93	(1.1–3.38)	535.23	0.56	0.042	1.164	(0.55–2.68)	0.356	0.126			0.854
≤ 35	96												
CgA (ng/mL)													
> 155	115	2.31	(1.38–3.89)	588.54	0.61	0.003	1.891	(0.86–4.14)	1.594	2.542			0.035
≤ 155	53												

When all variables identified as significant in the univariate analysis were included in a multivariate model, only CgA retained statistical significance (*p* = 0.035)

AIC = Akaike information criterion; C-index = Concordance index; CRP = C-reactive protein; HR = hazard ratio; NSE = neuron specific enolase; CgA = Chromogranin A; 95% CI = 95% confidence interval; *. Benjamini-Hochberg adapted p-value

**Table 3:** Multivariate analysis including at least three variables with the highest C-Index and lowest AIC according to OS

**A. Including Krenning score, hemoglobin and NSE (C-Index: 0.64; AIC 527.60)**				
**Variable**	**Multivariate Analysis**			
	**HR**	**(95% CI)**	**z.Score**	**P**
Krenning				
3	1.832	(1.08–3.12)	2.226	0.026
4				
Hemoglobin (g/dL)				
≤ 12	2.471	(1.35–4.54)	2.917	0.004
> 12				
NSE (ng/mL)				
> 35	2.014	(1.14–3.56)	2.414	0.016
≤ 35				
**B. Including BMI, hemoglobin and CgA (C-Index: 0.66; AIC 582.82)**				
**Variable**	**Multivariate Analysis**			
	**HR**	**(95% CI)**	**z.Score**	**P**
Body mass index				
≤ 18.5	2.291	(1.07–4.93)	2.123	0.034
> 18.5				
Hemoglobin (g/dL)				
< 12	2.078	(1.1–3.91)	2.262	0.024
≥ 12				
CgA (ng/mL)				
> 155	2.179	(1.29–3.68)	2.916	0.004
≤ 155				

A: AIC = Akaike information criterion; C-index = Concordance index; HR = hazard ratio; NSE = neuron specific enolase; 95% CI = 95% confidence interval

B: AIC = Akaike information criterion; C-index = Concordance index; CgA = Chromogranin A; 95% CI = 95% confidence interval; HR = hazard ratio

### Multivariate analysis

When all variables identified as significant in univariate analysis were included in a multivariate Cox regression model, only CgA remained statistically significant (*p* = 0.035; Table 2). None of the models including four or more covariates retained significance across all variables. When performing the integrated analysis of combinations of three variables, five different models yielded predictors of OS. Among these, two models demonstrated notably higher concordance with OS than the remaining three. The multivariate Cox regression model including KS, hemoglobin, and NSE showed the lowest AIC (C-index = 0.64, CI: 0.57–0.71; AIC = 527.60), while the model incorporating BMI, hemoglobin, and CgA demonstrated the highest C-index (C-index = 0.66, CI: 0.60–0.72) but a higher AIC of 582.82 (Table 3). Analysis of deviance showed that both multivariable models fit the data significantly better than the corresponding univariable models. All adjusted generalized variance inflation factor values were low, with a maximum adjusted GVIF of approximately 1.30, indicating no relevant multicollinearity (supplementary Table 1). As a secondary sensitivity analysis, LASSO-penalized Cox regression was performed to evaluate the robustness of variable selection and reduce the risk of overfitting. The LASSO model retained low hemoglobin, low BMI, elevated CgA, low albumin, Ki-67 > 5%, and age, with an apparent C-index of 0.710. Thus, the penalized model retained several variables that were also included in the main parsimonious models, including hemoglobin, BMI, and CgA. Krenning score and NSE were not retained in the LASSO model (supplementary Table 2).

### Baseline risk score for overall survival

**Fig. 2:**
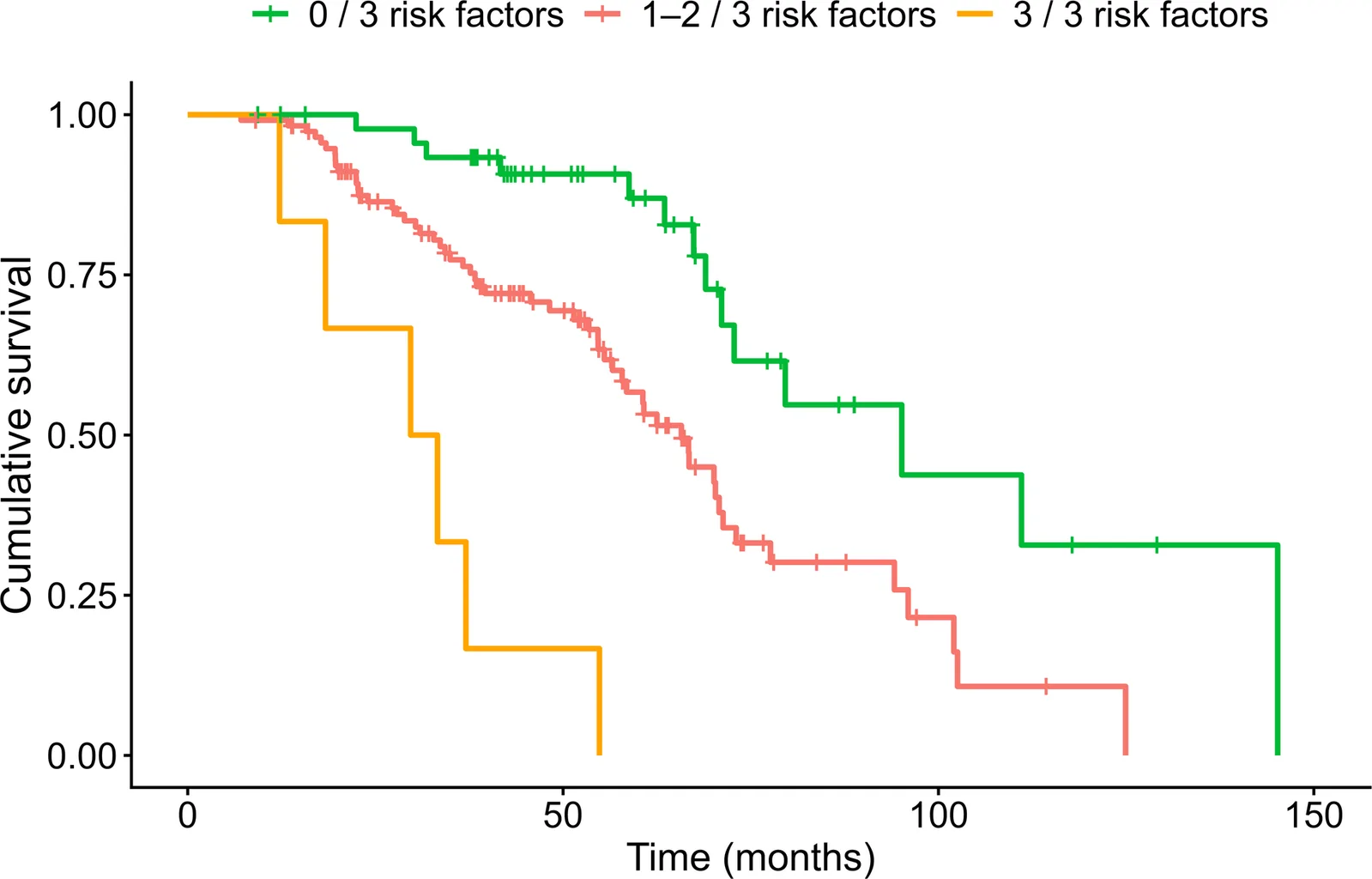
Kaplan–Meier overall survival curves stratified by baseline risk burden (0/3, 1–2/3, 3/3) derived from the integrated model including Krenning score, hemoglobin and neuron-specific enolase, demonstrating a stepwise decrease in median overall survival with increasing number of risk factors

**Fig. 3:**
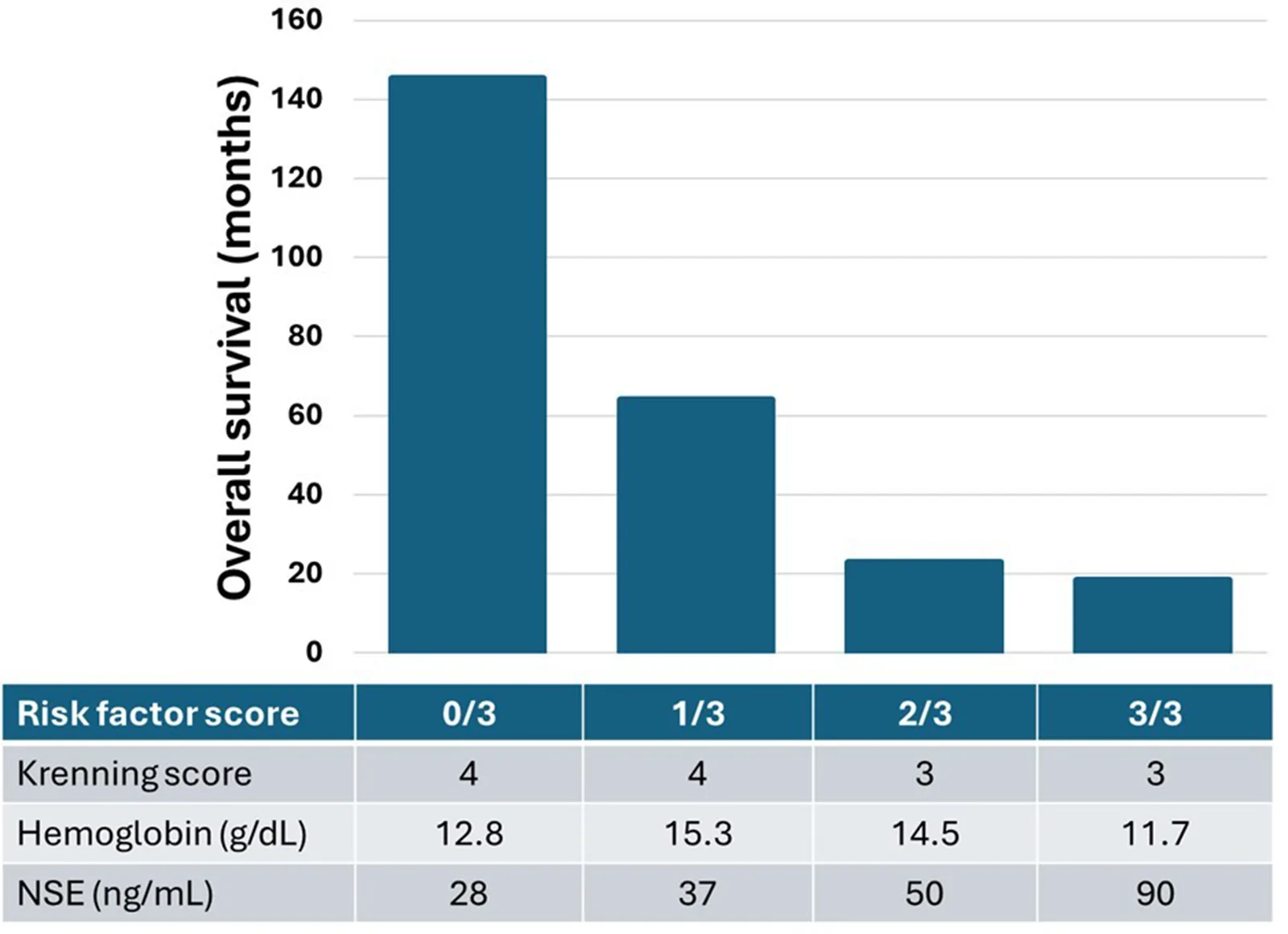
Exemplar patients illustrating the baseline risk score. Increasing numbers of risk factors were associated with progressively shorter overall survival

To facilitate visualization of risk stratification and enhance clinical usability, we translated the three-variables model including KS into a simple baseline risk score by assigning one point to each adverse factor (KS < 4, Hb < 12 g/dL, and NSE > 35 ng/mL), yielding a 0–3 score. Patients were grouped by the number of risk factors (0/3, 1–2/3, 3/3) and displayed as Kaplan–Meier curves (Fig. 2), showing a stepwise decrease in median OS from 95.1 months (0/3) to 66.7 months (1–2/3) and 31.5 months (3/3). Patients with 1–2 risk factors had significantly shorter OS than those with 0 risk factors (HR 2.29, 95% CI 1.27–4.14; *p* = 0.0059). Patients with 3 risk factors had significant shorter OS than both the 0 risk-factor group (HR 18.69, 95% CI 5.55–62.90; *p* < 0.001) and the 1–2 risk-factor group (HR 5.32, 95% CI 2.22–12.74; *p* < 0.001). To evaluate the influence of equal weighting, the simple additive score was compared with a coefficient-weighted Cox-derived score. The C-index was very similar for the equal-weight and coefficient-weighted scores (0.635 vs. 0.640), suggesting comparable discriminative performance while preserving the simplicity and interpretability of the equal-weight approach (supplementary Table 3). To further illustrate the clinical rationale underlying this score, representative patient examples with increasing numbers of risk factors are provided (Fig. 3). The illustrated example cases demonstrate a marked stepwise decline in overall survival with increasing risk burden, with OS times of 145.2 months (0/3 risk factors), 64.0 months (1/3), 22.8 months (2/3), and 18.3 months (3/3), thereby exemplifying the clinical gradient captured by the score.

### Subgroup analyses

Prior chemotherapy was associated with shorter OS in univariable analysis (HR 1.63, 95% CI 1.01–2.61; *p* = 0.044), but was not independently associated with OS when included in the multivariable Cox models and did not significantly change the estimated effects of the investigated baseline biomarkers (supplementary Table 4).

In further subgroup analyses, Ki-67 > 5% was more frequent in pNET than in siNET (61.1% vs. 25.2%; *p* < 0.001), while most other investigated baseline variables, including BMI, hemoglobin, albumin, CgA, NSE, and Krenning score distribution, did not differ significantly. Median OS was numerically shorter in pNET than in siNET (38.1 vs. 51.7 months), without reaching statistical significance in Kaplan-Meier analysis (log-rank *p* = 0.546) or univariable Cox regression (HR 1.20, 95% CI 0.67–2.15; *p* = 0.545). Interaction analyses provided no statistical evidence that the prognostic associations of the investigated baseline biomarkers differed between pNET and siNET, and adding primary tumor subgroup to the multivariable models did not significantly change model discrimination (C-Index = 0.650 and 0.665; supplementary Tables 5 & 6).

## Discussion

In this retrospective study of 178 patients with well-differentiated GEP-NET under PRRT, our findings support the hypothesis that integrating molecular imaging biomarkers, represented by the KS, with routinely available clinical and laboratory parameters may contribute to baseline risk stratification for OS prior to PRRT initiation. While several individual parameters demonstrated prognostic significance in univariate analysis, incorporation of the visually assessed KS provided complementary prognostic information in an integrated multivariate model, supporting the potential incremental value of molecular imaging within baseline risk stratification.

### Added value of molecular imaging

Patients with KS 3 compared to KS 4 had significantly shorter OS. These observations align with previous findings that higher in vivo tumor SSTR expression reflects a more differentiated tumor phenotype and corresponds to higher therapeutic uptake and response [20, 28–31]. Accordingly, current efforts to improve outcomes focus on dosimetry-guided PRRT individualization, such as higher cumulative activity or additional cycles within kidney/bone-marrow limits or strategies with SSTR antagonists are being investigated [32–35]. Recent evidence further supports the prognostic relevance of quantitative dosimetry in PRRT, particularly with regard to progression-free survival in GEP-NET patients [36]. However, the present study focused on routinely available baseline biomarkers before PRRT rather than treatment-delivered dose metrics. Future studies should investigate whether baseline integrated biomarkers and individualized dosimetry can be combined to further refine risk stratification.

More importantly, KS provided added value within integrated models. To our knowledge, integrated baseline models explicitly incorporating the routinely used visual Krenning score in PRRT-treated NET patients have been lacking. By showing that KS can be incorporated into a parsimonious multivariable model together with laboratory biomarkers, our findings suggest that molecular imaging may represent a valuable component of integrated diagnostic approaches in theranostic oncology.

### Biochemical and histopathological biomarkers and their association with OS

Several baseline biochemical and clinical markers were associated with overall survival, largely in line with previous literature in PRRT-treated NET patients. Elevated NSE, low hemoglobin, low BMI, and increased CRP have all been described as adverse prognostic indicators and showed expected associations with shorter OS in our cohort [37–39].

CgA was associated with shorter OS in both univariate and multivariate models at higher concentrations (> 155 ng/mL). These results align with findings from other studies indicating the prognostic value of CgA [20, 40–42]. However, other investigations, such as the NETTER-1 trial, have questioned the consistency of CgA as a prognostic marker in the PRRT setting [6, 38]. Variability in assay platforms, cutoffs, and confounding factors such as renal impairment or proton-pump inhibitor use may explain these discrepancies [43–47]. Despite ongoing debate, the strong performance of CgA in our large cohort underscores the need for further validation in prospective multicenter studies. This is also consistent with consensus recommendations emphasizing the clinical relevance of CgA and NSE while acknowledging limitations of currently available monoanalyte biomarkers in NET disease [13].

Hypoalbuminemia (albumin < 4.1 g/dL) was associated with poorer OS, consistent with prior studies identifying albumin and liver-related parameters as prognostic factors in PRRT-treated NET patients [42, 48]. These findings highlight the need for future studies to further investigate the prognostic impact of liver involvement in PRRT-treated NET patients, potentially combining further imaging-based and biochemical markers for refined risk stratification.

In contrast, Ki-67 was not strongly associated with OS, despite its established prognostic role [28, 38, 49]. The median 23-month interval between initial Ki-67 assessment and PRRT baseline, combined with known temporal and intratumoral heterogeneity of GEP-NET [50, 51], may explain this lack of association.

In subgroup analyses, Ki-67 > 5% was more frequent in pNET than in siNET, consistent with a biologically more aggressive phenotype in the pNET subgroup. However, interaction analyses provided no statistical evidence that the prognostic associations of the investigated baseline biomarkers differed between pNET and siNET. These subgroup findings should be interpreted cautiously due to the limited size and number of events in the pNET subgroup.

### Towards an integrated baseline risk stratification

Together, our data support the concept of a baseline assessment that merges molecular imaging, clinical biomarkers, and laboratory data to inform personalized treatment decisions. Importantly, the visual KS remains a practical tool that does not require labor-intensive post-processing or whole-body segmentation, enhancing its clinical utility and implementation [52]. Further, as a semi-quantitative visual scoring system, the KS allows standardized interpretation and facilitates comparability across different SSTR-targeting radiotracers and imaging protocols [5, 53]. The ease of acquiring these parameters, most of which are standard in NET diagnostic workup, facilitates their adoption in routine clinical workflows. Moreover, the intuitive structure of the derived risk score, assigning one point per adverse factor, could enable rapid bedside risk estimation, further supporting pragmatic and transparent patient stratification [54]. However, this risk score should be regarded as exploratory. Although comparison with a coefficient-weighted Cox-derived score showed similar discriminative performance, the score was derived and evaluated in the same retrospective cohort and may therefore be affected by optimism bias. Independent validation is required before considering routine clinical implementation.

Future prospective trials should explore whether integrating additional quantitative PET metrics, such as standardized uptake values (SUV) or volumetric tumor burden, could further refine survival prediction [55–57]. Looking ahead, AI-based clinical decision support could help model nonlinear interactions between routinely available parameters and automate parts of risk stratification, thereby facilitating their integration into everyday clinical workflows. However, such approaches should not be pursued as an end in themselves, and broader implementation will require robust prospective validation and careful integration into clinical infrastructure [54, 58, 59].

### Limitations

This study is limited by its retrospective design, single-center setting, and potential for selection bias. The statistical analyses were exploratory rather than prespecified model development. The combination of data-driven cut-point optimization, multiple model testing, and multivariable model selection may increase the risk of overfitting, particularly given the cohort size and number of events. Although Benjamini-Hochberg correction, multicollinearity assessment, LASSO-penalized Cox regression, and comparison of equal-weight and coefficient-weighted scores were added as complementary analyses, these approaches do not replace prospective external validation. Also, the data-derived thresholds should be interpreted cautiously, although several derived thresholds were close to commonly used clinical or laboratory reference values. The identified thresholds should therefore be considered exploratory and hypothesis-generating. Given the retrospective single-center design and limited cohort size, robust internal validation was not pursued as a separate model-development step, and external validation in independent, ideally multicenter cohorts is needed.

Follow-up duration, though adequate for survival estimation, may not capture late events or long-term effects beyond the initial PRRT course, such as salvage PRRT or other therapy options. Although additional adjustment for prior chemotherapy did not substantially change the estimated effects of the investigated baseline biomarkers, not all treatment-related confounders, including subsequent systemic therapies and salvage approaches, could be incorporated because of retrospective data and limited event numbers. Although we focused on patients receiving PRRT, future analyses should assess dynamic biomarkers over time to capture distinct response trajectories. Subgroup analyses comparing pNET and siNET were limited by the smaller pNET subgroup and limited number of events. Although no statistical evidence for subgroup-specific differences in the prognostic value of the investigated biomarkers was found, these analyses should be regarded as exploratory. Adequately powered studies are needed to further evaluate whether the prognostic value of integrated baseline biomarkers differs between GEP-NET entities. A potential drawback of this study is the application of multiple SSTR-specific radiotracers for PET imaging, including [⁶⁸Ga]Ga-DOTA-TATE, [⁶⁸Ga]Ga-DOTA-TOC, [⁶⁸Ga]Ga-DOTA-NOC, and [¹⁸F]SiTATE. While these radiotracers are known to offer comparable diagnostic reliability in clinical use [5, 60–62], they exhibit subtle differences in receptor subtype selectivity and distribution profiles, which may introduce variability in image assessment. Despite this, such variations are generally considered negligible and are not expected to have a substantial impact on the semi-quantitative measurement of KS, which remains robust across the tracers employed [5, 60, 63, 64]. Finally, we did not validate our integrated model in an external dataset, which may limit generalizability. Future prospective studies should evaluate the additive value of imaging biomarkers in combination with biochemical and clinical variables, ideally in externally validated, multi-institutional cohorts.

## Conclusions

While several baseline parameters were associated with overall survival in univariate analyses, multivariate modeling identified specific integrated combinations of clinical, laboratory, and imaging biomarkers that may improve baseline risk stratification. Incorporation of the routinely available KS from SSTR-PET provided additional prognostic value at the model level, suggesting the possible contribution of molecular imaging biomarkers within integrated diagnostic approaches for baseline risk stratification and personalized theranostic management in GEP-NET patients undergoing PRRT. Given the retrospective and exploratory nature of this study, the robustness and generalizability of these findings require validation in larger, preferably multicenter, independent cohorts before consideration for clinical implementation.

## Supplementary Information

Below is the link to the electronic supplementary material.

Supplementary Material 1

## Data Availability

The datasets used and/or analyzed during the current study are not publicly available due to institutional data protection regulations and patient privacy restrictions, but are available from the corresponding author on reasonable request and subject to institutional approval.

## Abbreviations

AI: Artificial intelligence
AIC: Akaike information criterion
ANOVA: Analysis of variance
BMI: Body mass index
CgA: Chromogranin A
CI: Confidence interval
C-index: Concordance index
CRP: C-reactive protein
CT: Computed tomography
DOTA-NOC: DOTA-1-Nal3-octreotide
DOTA-TATE: DOTA-Tyr3-octreotate
DOTA-TOC: DOTA-Tyr3-octreotide
ENETS: European Neuroendocrine Tumor Society
GEP-NET: Gastroenteropancreatic neuroendocrine tumor
Hb: Hemoglobin
HR: Hazard ratio
KS: Krenning score
MAR: Missing at random
MBq: Megabecquerel
MDT: Multidisciplinary tumor board
NANETS: North American Neuroendocrine Tumor Society
NET: Neuroendocrine tumor
NSE: Neuron-specific enolase
OS: Overall survival
PET: Positron emission tomography
PFS: Progression-free survival
PRRT: Peptide receptor radionuclide therapy
SSTR: Somatostatin receptor
SUV: Standardized uptake value
