# Supplementary material for: Krenning score enhances integrated biomarker models for survival prediction in PRRT-treated GEP-NET: a retrospective cohort study
Source: Cancer Imaging. 2026 Aug 25;26:112. doi: 10.1186/s40644-026-01113-w (PMC13505048; doi:10.1186/s40644-026-01113-w)
Supplement: Supplementary file 1 [file 40644_2026_1113_MOESM1_ESM.docx]

**Supplementary Table 1.** Multicollinearity analysis of investigated baseline predictors. All adjusted GVIF values were low, with a maximum adjusted GVIF of 1.304, indicating no relevant multicollinearity among the investigated baseline variables.

| **Variable** | **GVIF** | **Adjusted GVIF** |
| --- | --- | --- |
| Krenning score | 1.319 | 1.148 |
| BMI | 1.403 | 1.184 |
| Hemoglobin | 1.700 | 1.304 |
| NSE | 1.207 | 1.099 |
| CgA | 1.208 | 1.099 |
| CRP | 1.370 | 1.170 |
| Albumin | 1.561 | 1.249 |
| Ki-67 | 1.449 | 1.204 |
| Prior chemotherapy | 1.448 | 1.204 |
| Primary tumor group | 1.507 | 1.108 |
| BMI = body mass index; CgA = chromogranin A; CRP = C-reactive protein; GVIF = generalized variance inflation factor; NSE = neuron-specific enolase. | | |

**Supplementary Table 2.** LASSO-penalized Cox regression sensitivity analysis for overall survival. The penalized model retained low hemoglobin, low BMI, elevated CgA, low albumin, Ki-67 >5%, and age, with an apparent C-index of 0.710 at lambda min. Krenning score and NSE were not retained in the penalized model.

| **Variable retained by LASSO** | **Coefficient** | **HR** |
| --- | --- | --- |
| Hemoglobin ≤12 g/dL | 0.214 | 1.238 |
| BMI ≤18.5 kg/m² | 0.445 | 1.560 |
| CgA >155 ng/mL | 0.270 | 1.310 |
| Albumin <4.1 g/dL | 0.195 | 1.216 |
| Ki-67 >5% | 0.004 | 1.004 |
| Age at baseline | 0.015 | 1.015 |
| BMI = body mass index; CgA = chromogranin A; C-index = concordance index; HR = hazard ratio; LASSO = least absolute shrinkage and selection operator; NSE = neuron-specific enolase | | |

**Supplementary Table 3.** Cox regression analysis of the coefficient-weighted score. The additive equal-weight score assigned one point to each adverse predictor, whereas the coefficient-weighted score was derived from Cox regression coefficients. The coefficient-weighted score showed a comparable C-index to the additive equal-weight score reported in the main manuscript (0.640 vs. 0.635).

| **Coefficient-weighted score (AIC = 530.2, C-index 0.640)** | | | | |
| --- | --- | --- | --- | --- |
| **Term** | **HR** | **95% CI** | **p-value** |  |
| 1–2 risk factors vs. 0 risk factors | 1.207 | 0.644–2.264 | 0.557 |  |
| 3 risk factors vs. 0 risk factors | 2.677 | 1.475–4.856 | 0.001 |  |
| AIC = Akaike information criterion; C-index = concordance index; CI = confidence interval; HR = hazard ratio | | | |  |

**Supplementary Table 4.** Multivariable Cox regression models additionally adjusted for prior chemotherapy. Prior chemotherapy was not independently associated with OS when included in the multivariable Cox models and did not substantially change the estimated effects of the investigated baseline biomarkers.

**A.** Model including Krenning score, hemoglobin, NSE, and prior chemotherapy (C-Index: 0.653; AIC 529.5).

| **Variable** | **HR** | **95% CI** | **p-value** | |
| --- | --- | --- | --- | --- |
| Krenning score 3 vs. 4 | 1.806 | 1.044–3.123 | 0.035 | |
| Hemoglobin ≤12 g/dL | 2.433 | 1.305–4.534 | 0.005 | |
| NSE >35 ng/mL | 2.008 | 1.136–3.547 | 0.016 | |
| Prior chemotherapy | 1.062 | 0.630–1.792 | 0.820 | |
| AIC = Akaike information criterion; C-index = concordance index; CI = confidence interval; HR = hazard ratio; NSE = neuron-specific enolase | | | |  |
|  | | | |  |

**B.** Model including BMI, hemoglobin, CgA, and prior chemotherapy (C-Index: 0.645; AIC 584.6).

| **Variable** | **HR** | **95% CI** | **p-value** | |
| --- | --- | --- | --- | --- |
| BMI ≤18.5 kg/m² | 2.333 | 1.079–5.043 | 0.031 | |
| Hemoglobin ≤12 g/dL | 1.999 | 1.037–3.855 | 0.039 | |
| CgA >155 ng/mL | 2.135 | 1.256–3.631 | 0.005 | |
| Prior chemotherapy | 1.123 | 0.668–1.889 | 0.661 | |
| AIC = Akaike information criterion; BMI = body mass index; CgA = chromogranin A; C-index = concordance index; CI = confidence interval; HR = hazard ratio | | | |  |

**Supplementary Table 5.** Subgroup-specific univariable Cox regression analyses. Statistical significance was mainly observed in the larger siNET subgroup, while several estimates in pNET showed similar directions but wider confidence intervals, likely reflecting the smaller number of patients and events.

|  | **siNET** | | | | **pNET** | | | | |
| --- | --- | --- | --- | --- | --- | --- | --- | --- | --- |
| **Variable** | **HR** | **95% CI** | **p-value** | **C-index** | **HR** | **95% CI** | **p-value** | **C-index** | |
| BMI ≤18.5 kg/m² | 3.428 | 1.447–8.121 | 0.005 | 0.551 | 1.001 | 0.128–7.803 | 1.000 | 0.473 | |
| Krenning score 3 vs. 4 | 1.785 | 0.948–3.363 | 0.073 | 0.554 | 1.471 | 0.404–5.358 | 0.558 | 0.556 | |
| Ki-67 >5% | 1.281 | 0.681–2.413 | 0.442 | 0.523 | 1.789 | 0.586–5.464 | 0.307 | 0.575 | |
| CRP >0.5 mg/dL | 2.190 | 0.952–5.037 | 0.065 | 0.569 | 1.380 | 0.375–5.083 | 0.628 | 0.584 | |
| Albumin <4.1 g/dL | 1.761 | 0.889–3.489 | 0.105 | 0.565 | 3.350 | 0.961–11.683 | 0.058 | 0.642 | |
| Hemoglobin ≤12 g/dL | 2.253 | 1.084–4.686 | 0.030 | 0.559 | 2.449 | 0.519–11.542 | 0.258 | 0.553 | |
| NSE >35 ng/mL | 1.855 | 0.907–3.796 | 0.091 | 0.563 | 2.341 | 0.712–7.697 | 0.161 | 0.586 | |
| CgA >155 ng/mL | 2.745 | 1.367–5.512 | 0.005 | 0.610 | 2.354 | 0.730–7.583 | 0.152 | 0.618 | |
| BMI = body mass index; CgA = chromogranin A; CI = confidence interval; C-index = concordance index; CRP = C-reactive protein; HR = hazard ratio; NSE = neuron-specific enolase; pNET = pancreatic neuroendocrine tumor; siNET = small intestinal neuroendocrine tumor | | | | | | | | |  |

**Supplementary Table 6.** Interaction analyses for primary tumor site. Cox models with and without interaction terms between primary tumor site and each investigated biomarker were compared. Interaction p-values >0.05 and higher AIC values in the interaction models indicated no statistical evidence for subgroup-specific differences in prognostic associations between pNET and siNET.

| **Variable** | **AIC without interaction** | **AIC with interaction** | **Interaction p-value** | |
| --- | --- | --- | --- | --- |
| BMI | 542.9 | 543.5 | 0.231 | |
| Krenning score | 543.8 | 545.7 | 0.731 | |
| Ki-67 | 545.4 | 547.1 | 0.543 | |
| CRP | 381.4 | 382.0 | 0.232 | |
| Albumin | 414.6 | 414.9 | 0.195 | |
| Hemoglobin | 541.9 | 543.6 | 0.581 | |
| NSE | 450.8 | 452.4 | 0.528 | |
| CgA | 492.3 | 494.3 | 0.930 | |
| AIC = Akaike information criterion; BMI = body mass index; CgA = chromogranin A; CRP = C-reactive protein; NSE = neuron-specific enolase; pNET = pancreatic neuroendocrine tumor; siNET = small intestinal neuroendocrine tumor | | | |  |
